# Can Winter-Active Bumblebees Survive the Cold? Assessing the Cold Tolerance of *Bombus terrestris audax* and the Effects of Pollen Feeding

**DOI:** 10.1371/journal.pone.0080061

**Published:** 2013-11-05

**Authors:** Emily L. Owen, Jeffrey S. Bale, Scott A. L. Hayward

**Affiliations:** School of Biosciences, University of Birmingham, Birmingham, United Kingdom; University of Arizona, United States of America

## Abstract

There is now considerable evidence that climate change is disrupting the phenology of key pollinator species. The recently reported UK winter activity of the bumblebee *Bombus terrestris* brings a novel set of thermal challenges to bumblebee workers that would typically only be exposed to summer conditions. Here we assess the ability of workers to survive acute and chronic cold stress (via lower lethal temperatures and lower lethal times at 0°C), the capacity for rapid cold hardening (RCH) and the influence of diet (pollen versus nectar consumption) on supercooling points (SCP). Comparisons are made with chronic cold stress indices and SCPs in queen bumblebees. Results showed worker bees were able to survive acute temperatures likely to be experienced in a mild winter, with queens significantly more tolerant to chronic cold temperature stress. The first evidence of RCH in any Hymenoptera is shown. In addition, dietary manipulation indicated the consumption of pollen significantly increased SCP temperature. These results are discussed in the light of winter active bumblebees and climate change.

## Introduction

Climate change has resulted in changes to the physiology, survival, abundance and range of many organisms [1]. Characterisation of these changes is essential to understanding and mitigating the potential impacts of climate warming [2] and the provision of vital ecosystem services. This is particularly important in insect pollination [3] [4], with considerable evidence reporting phenology shifts in key pollinator species [5] [6] [7]. Spring advancement, extended growing seasons, milder winters and the availability of winter forage [1] have all combined to allow some normally univoltine species to become bivoltine, as evidenced by winter active bumblebees, *Bombus terrestris*, in the southern UK [8]. This raises a number of new challenges, in particular the survival of life stages previously unexposed to winter conditions.

At temperate latitudes, a specialised state of dormancy, termed diapause, is typically utilised as an overwintering strategy in virtually all insects [9] [10], and winter survival of cold stress outside of diapause is greatly reduced [11]. In bumblebees, as with many bee species, it is only mated females (queens) that enter diapause and overwinter, but we know very little about how bumblebee queens or other life stages (workers and males) cope with cold conditions. Diapause in bumblebees is not thought to be obligate, as there is evidence that commercial colonies can be established by queens without diapause [12]. There are also several examples of bivoltinism in Mediterranean field populations [13] [14], as well as in New Zealand and Tasmania [15]. In addition, non-diapause characteristics can be artificially selected for, and can persist across multiple generations [16]. Considerable flexibility also exists in the seasonal timing of diapause in *B. terrestris*, with both a summer aestivation and winter hibernation reported in different regions of Turkey [17]. The issue in the UK, and in other parts of Northern Europe, is that *B. terrestris* queens appear to be averting diapause, or have a greatly curtailed diapause, under warmer conditions and so are establishing new colonies in late Summer/Autumn [8]. This is in stark contrast to a previous study [18], that reported a period of dormancy spanning 6–9 months in the UK.

Certainly to assume climate change will reduce cold-induced winter mortality is over simplistic, as even under global warming conditions, winters at temperate latitudes will remain cold – indeed there is increasing evidence of more frequent extreme climatic events [19]. Key to the winter survival of insects is the magnitude, frequency and timing of cold events with variable thermal regimes conferring a reduction in chill-injury and increased longevity compared to constant low temperatures [20]. Thus, it is crucial to determine whether key pollinator species such as bumblebees will actually benefit from climate change, or if winter activity might have a negative impact on their abundance, distribution and pollination service provision. Addressing this knowledge gap is important from both conservation and food security perspectives, given the critical pollination services provided by bumblebees. *Bombus terrestris*, in particular, are key economic crop pollinators, essential in the pollination of vegetable, fruit and seed crops [21] and are responsible for the annual production of 40,000ha of tomato crops (*Solanum lycopersicum)* [22]. As a result of their success, commercially-produced bumblebee colonies are exported from Europe at a rate of 850,000 per year to Asia, North Africa, Chilie, New Zealand and the Middle East [23].

To survive winter, active colonies of the UK subspecies, *B. t. audax*, must first be able to withstand potential acute low temperatures when foraging, and chronic cold exposures within the colony. Bumblebee colonies are known to regulate their temperature to a set point between 27 and 33°C [24]. However, chronic low temperatures have been found to disrupt colony thermoregulation and have a negative impact on colony fecundity and brood incubation temperature [25]. With a reduced number of workers, smaller colonies are less able to respond to environmental stresses such as temperature fluctuations [26]. Acute cold exposure also poses a risk to individual bumblebees foraging in low daytime temperatures, or bees spending the night away from the nest [27] [28]. The response of workers to chronic and acute cold exposure is currently unknown, presenting an uncertain risk to the survival of winter-active colonies, and thus pollinator abundance the following spring.

Insects employ several mechanisms to increase their tolerance of chilling or cold shock, one of which is Rapid Cold Hardening (RCH). This is where a short period (e.g. 1 hour) of acclimation leads to significantly higher survival at sub-zero temperatures [29]. The ability to rapidly cold harden was first identified in the flesh fly (*Sarcophaga crassipalpis*), but has since been indentified in 8 orders and 26 families. Interestingly, RCH has not yet been recorded in any Hymenoptera [30]. In the case of winter-active *B. t. audax*, RCH is likely to have considerable adaptive significance in the survival of workers and active queens.

Gut contents are also known to play an important role in the cold tolerance of insects and their manipulation is often a discriminating factor between freeze tolerant and freeze avoiding strategies [31]. Freeze avoiding (FA) insects seek to physiologically reduce their freezing temperature/supercooling point (SCP), and are known to evacuate their guts to exclude ice nucleating agents (INAs) such as bacteria, dust and food particles [32] which might promote the formation of ice crystals [33]. Typical SCPs for FA insects are below −10°C [30] e.g. *Collembola* sp. with SCPs between −15 and −35°C [34]. However, freeze tolerant species actively produce INAs to facilitate more controlled freezing at higher sub-zero temperatures, and so typically have SCPs between −5 and −10°C e.g. overwintering larvae of *Eurosta solidaginis* with an SCP of −9°C [35]. Certainly gut contents can have a huge impact on an organism's SCP, and consequently the winter survival of FA species. For example, the absence of gut contents in starved, winter Antarctic microarthropods is responsible for a shift in SCP from a summer ‘high’ group (mean SCP −7°C) to a winter ‘low’ group (mean SCP −25°C) [36]. It is not known if *B. terrestris* is FT or FA, but we hypothesise this species will not tolerate freezing, in common with several other bee species, e.g. *Osmia cornuta* and *O. rufa*, both with SCPs typically below −24°C [37], and *Megachile rotundata* with SCPs of −8°C [38]. Given the ice nucleating properties of pollen [39] winter active, and thus feeding, workers, especially those remaining outside the colony at night, are at increased risk of mortality due to freezing. Winter active bumblebees (as described by [8]) forage for both nectar and pollen in order to provision their colonies. Currently, however, there does not appear to be any direct evidence that workers ingest pollen. If they do, this could negatively impact their survival at sub-zero temperatures. For example, the presence and quantity of ice nucleators in the gut was found to negatively impact cold tolerance in *M. rotundata* [40].

The objectives of this study were to assess the ability of *B. t. workers* to tolerate UK winter temperatures and compare their cold tolerance with active queens. This involved a determination of their capacity for RCH and survival following prolonged periods of cold exposure consistent with what might be experienced within winter colonies. An assessment of their freezing temperature (SCP) and post-freezing survival also permitted classification of this species as either ‘freeze tolerant’ or ‘freeze avoiding’, consistent with criteria outlined by [31]. Finally, the impact of diet on cold tolerance was investigated, to determine the effect of pollen on the cold tolerance of foraging bumblebees.

## Materials and Methods

### Culture system

Mature colonies of B. t. audax, were obtained from Biobest NV (Westerlo, Belgium), maintained at 20°C in constant darkness and manipulated under red illumination to minimise disturbance [41]. Nectar was available within the colony using a wick system connected to a reservoir of BioGluc® nectar and pollen paste was available ad libitum (Biobest NV). Mated, non-diapausing queens were also provided by Biobest NV (Westerlo, Belgium) and were individually housed in ‘feeding boxes’ containing pollen and nectar ad libitum (unless otherwise stated). For each experimental treatment, n = 30 bees were used, unless otherwise stated. Control samples of 30 bees were exposed to 15°C for the maximum experimental duration and survival, determined as the response to gentle manipulation, was assessed after 72 h.

### Lower lethal temperature

Bumblebees were placed in to 6 test tubes (*n* = 5 per tube) containing type K exposed wire thermocouples to record body temperature. Tubes were placed into an alcohol bath (Haake Phoenix 11 P2, Thermo Electron Corporation), programmed to cool from 20°C, at a rate of 0.2°Cmin^−1^, to a range of sub-zero temperatures. Bumblebees were held at each temperature for 15 min before the temperature was increased back to 20°C at the same rate. Bees were transferred to rearing conditions and survival assessed after 72 h.

### Lower lethal time at 0°C

Workers were added in groups of 5 to 6 conical flasks (25 ml Pyrex, *n* = 30 per treatment) and placed inside a Fryka® incubator set at 0°C for a range of durations. Before and after each exposure at 0°C, bees were held at 10°C for 1 h to prevent the possibility of cold and heat shock mortality respectively. Bumblebees were removed from the incubator, added to a recovery box and survival was assessed as previously described

Queens were added in groups of 5 to 3 conical flasks (100 ml Pyrex, *n* = 15 per treatment), manipulated in the same way as workers, and survival assessed as previously described.

### Rapid cold hardening

#### Determination of the discriminating treatment

Temperatures of −5 and −6°C were chosen as they represented the lowest sub-zero temperatures which induced mortality whilst having minimal incidence of freezing (supercooling point ranged from −5.0 to −10.9°C). Thirty bumblebees (6 replicates of *n* = 5 bees per temperature) were taken from their rearing temperature (20°C), added to test tubes with thermocouples as previously described, and placed directly in an alcohol bath set at −5 or −6°C for a range of durations. Bees were then re-warmed to rearing temperature at a rate of 0.2°Cmin^−1^ and survival was assessed after 72 h. A treatment that resulted in 10–20% survival at a particular time interval was used as the discriminating treatment [42].

#### Rapid cold hardening response

Thirty bumblebees (6 replicates of *n* = 5 bees per treatment) were added to test tubes as previously described and exposed to one of two temperature regimes: 1 h at 0°C before transfer to the discriminating treatment, re-warming at 0.2°Cmin^−1^, or gradual cooling at 0.2°Cmin^−1^ to the discriminating treatment, and re-warming at the same rate. Survival in both experiments was assessed after 72 h. RCH was detected as an increase in survival relative to direct transfer to the discriminating treatment.

#### Impact of rapid cold hardening on supercooling point

After a period of 1 h at 0°C, the supercooling points (SCPs) of 30 workers were measured, using established methods [43]. Briefly, bees were inserted individually into test tubes containing type K exposed wire thermocouples, placed in an alcohol bath programmed to cool from 20°C to −20°C at a rate of 0.2°Cmin^−1^ and freezing exotherms were detected via a computerized recording system.

### Impact of diet on gut pollen content and SCP

#### Pollen and nectar diet

Workers (*n* = 30) and queens (*n* = 21) were removed from their colonies and feeding boxes respectively and their SCPs measured as previously described.

#### Nectar-only diet

Workers were kept in ‘feeding boxes’ and fed nectar-only for a period of 3, 7 or 14 days before their SCPs were measured (*n* = 30 per duration). Queens were kept individually in feeding boxes and fed nectar-only for 7 days (limited numbers meant only this time point could be tested), before their SCPs were measured (*n* = 20). Following SCP measurement, worker bumblebees maintained under the feeding treatments described above were dissected and their gut contents examined under a high power microscope (*n* = 16, 8 and 6 for 3, 7 and 14 day nectar-fed workers respectively). Gut contents were partitioned into 3 µl aliquots, and pollen grains were quantified in each sample. The total pollen count of each individual was then compared to the supercooling point of the bee and the days of nectar feeding.

### Statistical analysis

All results were tested for normality using a Kolmogorov-Smirnov test. LLTemp_10,50,90_ and LLTime_10,50,90_ experiments were analysed via Probit analysis [44] in Minitab® to identify the temperature or time at which 10, 50 or 90% mortality occurred. RCH and diet experiments were non-normally distributed and so independent samples Kruskal Wallis tests with pairwise comparisons were undertaken in SPSS®. RCH and SCP results were compared via a one way ANOVA in Minitab®. Regression analysis was undertaken on total pollen versus supercooling point data (with model checking to ensure normally distributed errors, a linear relationship and equal variance of errors) and proportions of *B. t. audax* workers with pollen grains in their guts were analysed by means of a Chi square test, all using Minitab®.

## Results

### Lower lethal temperature

**Figure 1:**
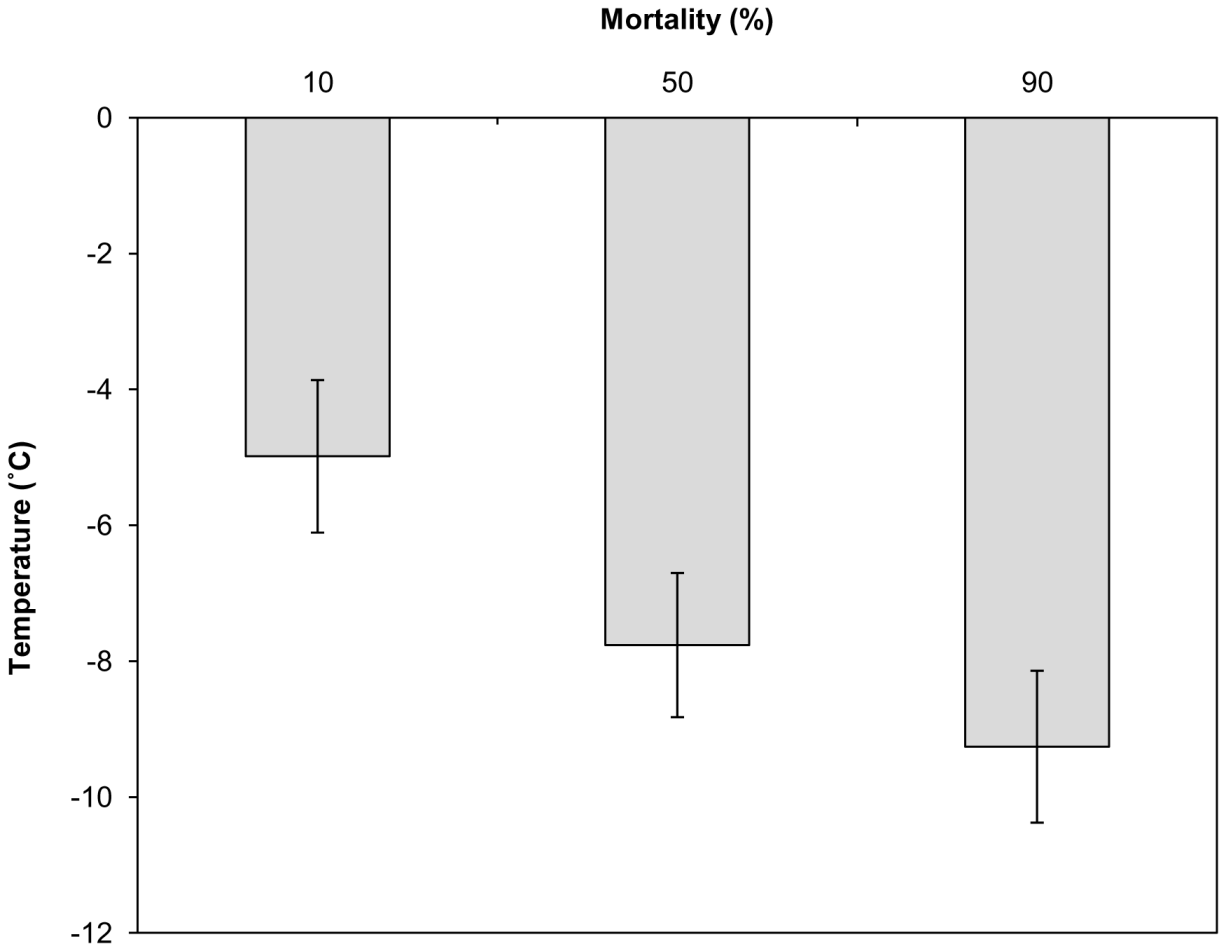
Lower Lethal Temperatures of worker bumblebees (*Bombus terrestris audax*). The LLTemp_10,50_ and LLTemp_90_ (±SE) of worker bumblebees as determined by Probit analysis, *n* = 30 per temperature.

Lethal temperatures for 10, 50 and 90% mortality of workers were −5.0±1.1, −7.8±1.1 and −9.3±1.1°C respectively (Figure 1). No mortality was recorded in control samples.

### Lower lethal time at 0°C

**Figure 2:**
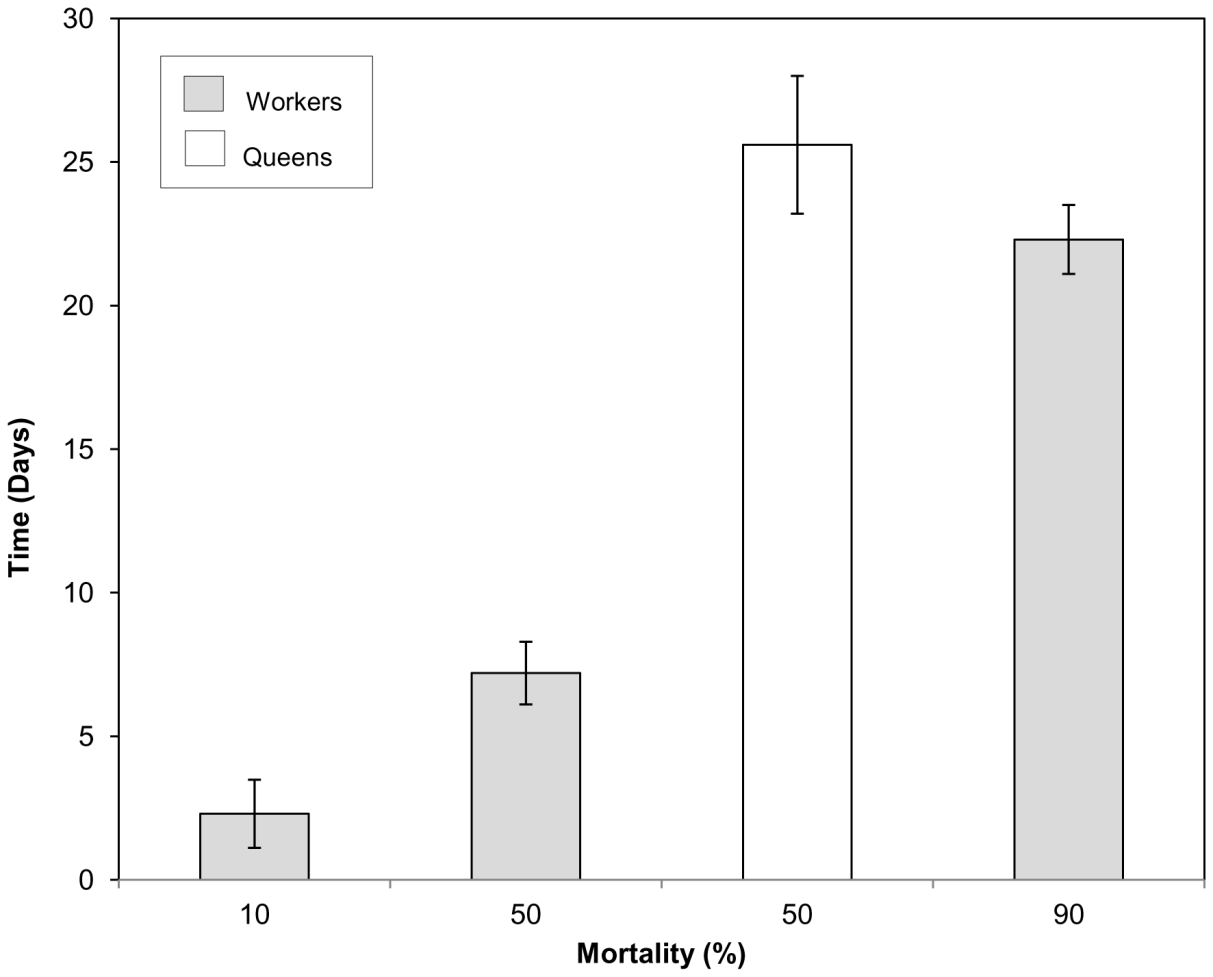
Lower Lethal Times at 0°C of worker and queen bumblebees (*Bombus terrestris audax*). The LLTime_10,50_ and LLTime_90_ at 0°C (±SE) of worker bumblebees as determined by Probit analysis, *n* = 30 per duration.

Lethal times for 10, 50 and 90% mortality at 0°C for workers were 2.3±1.2, 7.2±1.1 and 22.3±1.2 days respectively (Figure 2). The LLTime_50_ for queens at 0°C was 25.6±2.4 days, which was significantly different from that of workers (using non-overlapping fiducial limits; Hughes et al., 2009). As sample sizes were smaller for queens (*n* = 15) than workers (*n* = 30), only LLTime_50_ was determined. No mortality was recorded in control samples.

### Rapid cold hardening

#### Determination of the discriminating treatment

**Figure 3:**
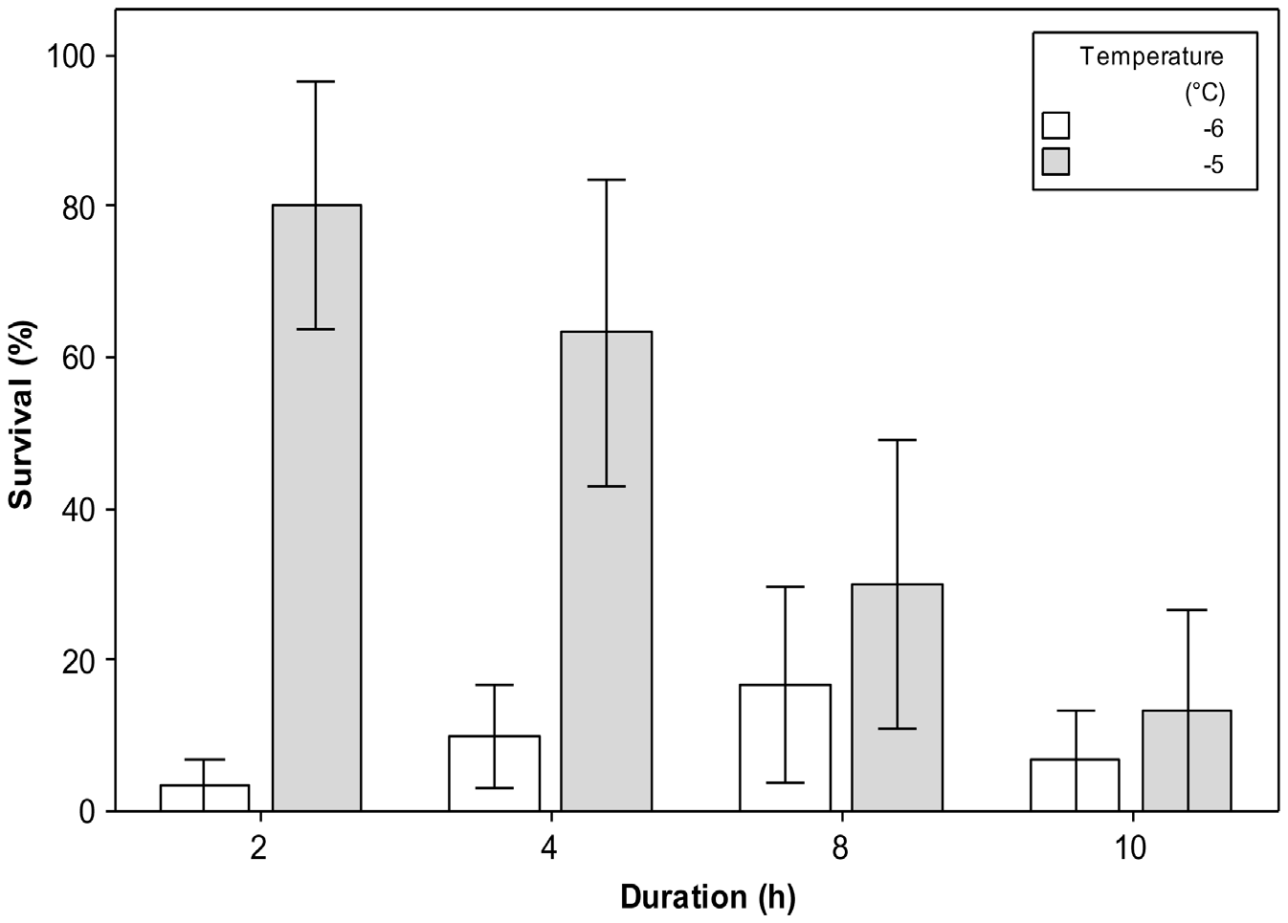
Determination of a discriminating treatment for rapid cold hardening in worker bumblebees (*Bombus terrestris audax*). Mean survival (±SE) of worker bumblebees exposed to periods of 2, 4, 8 and 10 h at −5 and −6°C, *n* = 30 per temperature.

Mean survival of workers at −5°C decreased with increasing duration of cold exposure, from 80±16.3% after 2 h to 13.3±13.3% after 10 h exposure (Figure 3). An independent samples Kruskal Wallis test (*p* = 0.03, χ = 8.66) with pairwise comparisons indicated survival at 2 and 10 h were significantly different (*p* = 0.045 χ = 9.92). Mean survival of workers at −6°C was consistently low (between 3.3±3.3 and 16.7±13.1% survival) with no significant difference between durations at this temperature (*p* = 0.19, χ = 0.93). Based on the above data, 10 h at −5°C was chosen to be the discriminating treatment to determine a RCH response.

#### Rapid cold hardening response

**Figure 4:**
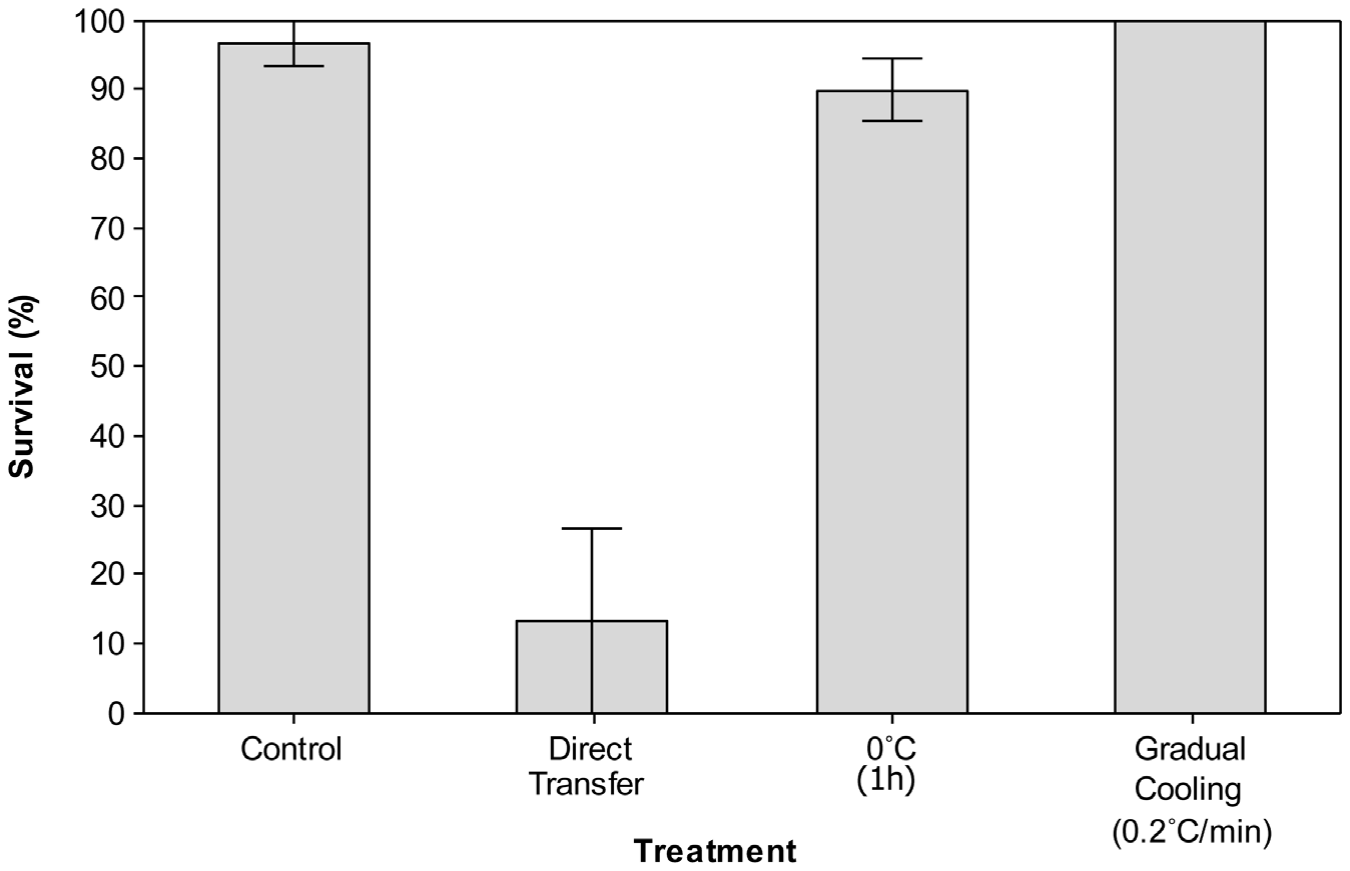
A rapid cold hardening response in worker bumblebees (*Bombus terrestris audax*). Mean survival (±SE) of worker bumblebees after direct transfer to the discriminating treatment (−5°C for 10 h), a pretreatment of 1 h at 0°C or gradual cooling (at 0.2 min^−1^) to the discriminating treatment. *n* = 30 per treatment.

Survival of workers gradually cooled at 0.2 min^−1^ to the discriminating treatment, (100±0%) was significantly higher (*p* = 0.002, *χ* = −13.17) than survival as a result of direct transfer (13.3±13.3%; Figure 4). Survival following a 1 h pretreatment at 0°C before transfer to the discriminating treatment (90.0±4.47%) proved non-significant (*p* = 0.08, *χ* = −8.67).

#### Rapid cold hardening and supercooling point

The supercooling points of workers exposed to a pretreatment of 8 h at 0°C were not significantly different from controls (Table 1) (*F* = 1.12, *p* = 0.48).

**Table 1:** Affect of rapid cold hardening on supercooling point in *Bombus terrestris audax*.

Treatment group	*n*	Mean±SE°C	Range °C
SCP control	30	−7.1±0.2	−5.0 to −10.9
SCP with RCH	30	−7.3±0.2	−5.62 to −12.8

Supercooling points (SCP) of *Bombus terrestris audax* with no pretreatment (control) and a pretreatment at 0°C for 1 h (RCH), *n* = 30.

### Impact of diet on gut pollen content and SCP

**Figure 5:**
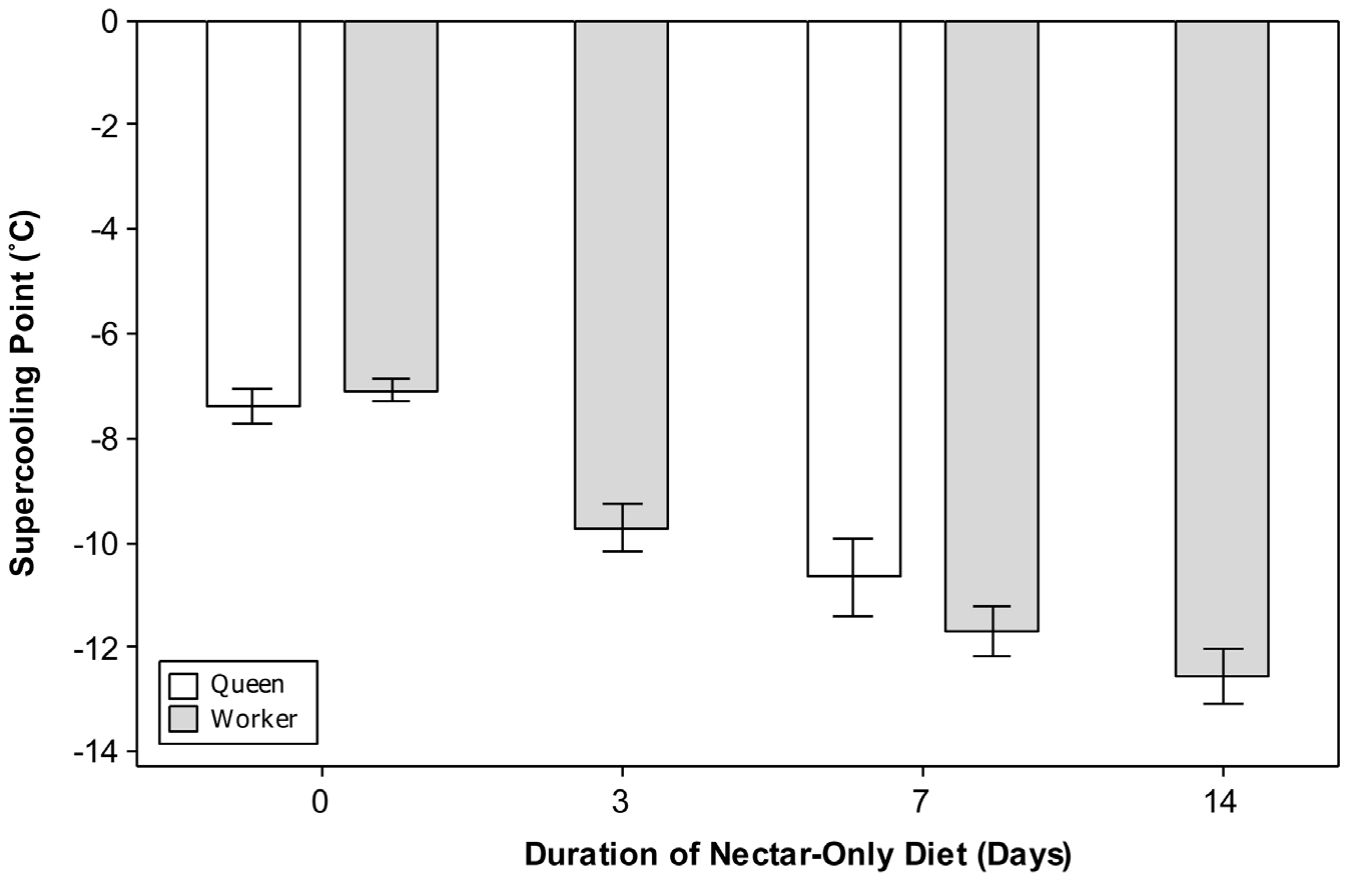
The affect of diet on supercooling point in queen and worker bumblebees (*Bombus terrestris audax*). Mean supercooling points (±SE) of *Bombus terrestris audax* workers and queens when fed a nectar-only diet for 3, 7 or 14 days with pollen and nectar-fed controls (*n* = 30 for all worker treatments and *n* = 20 and 19 for queen controls and 7 day nectar-fed-queens respectively).

Preliminary experiments indicated both workers and queens suffered 100% mortality as a result of freezing. The mean SCP of workers decreased from −7.1±0.2°C in controls to −9.7±0.5, −11.7±0.5 and −12.5±0.5 after 3, 7 and 14 days of nectar-only feeding respectively (Figure 5). Each nectar-only feeding group had a significantly lower SCP than controls (*p*≤0.01 in all cases) Only the 3 and 14 day groups were significantly different from each other (*p* = 0.03, *χ* = 37.57). The mean SCP of queens significantly decreased from −7.4±0.3°C in controls to −10.6±0.7°C after 7 days of nectar feeding (*p* = 0.01 *χ* = 50.58). The SCPs of workers and queens were never significantly different from each other (*p>*0.05).

**Figure 6:**
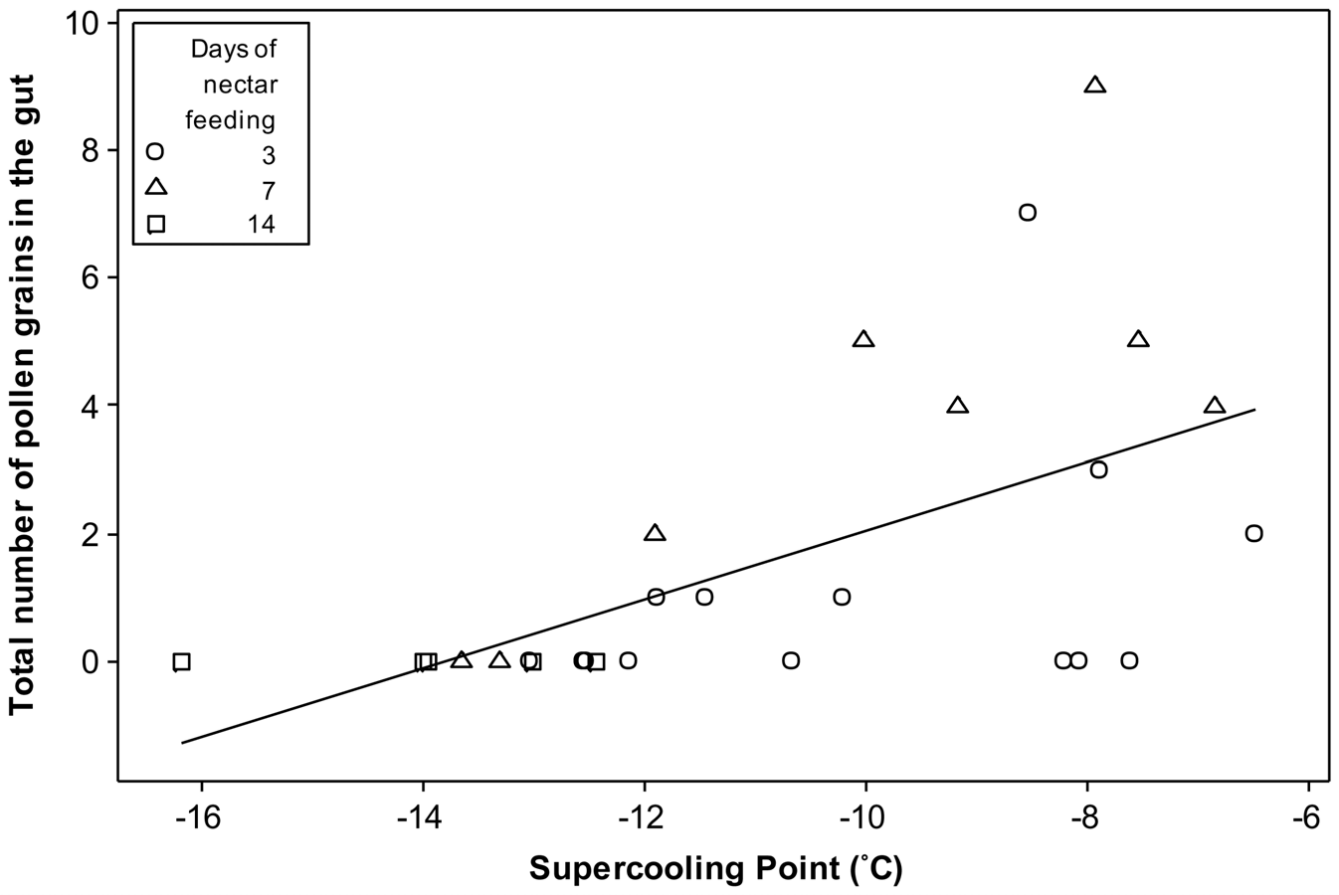
The affect of dietary pollen on supercooling point. Total number of pollen grains found in the guts of *Bombus terrestris audax* workers, plotted against their corresponding supercooling points (*n* = 14, 8 and 5 for 3, 7 and 14 day nectar-fed workers respectively), a significant relationship (R^2^ = 32.7%, *p*<0.01).

**Figure 7:**
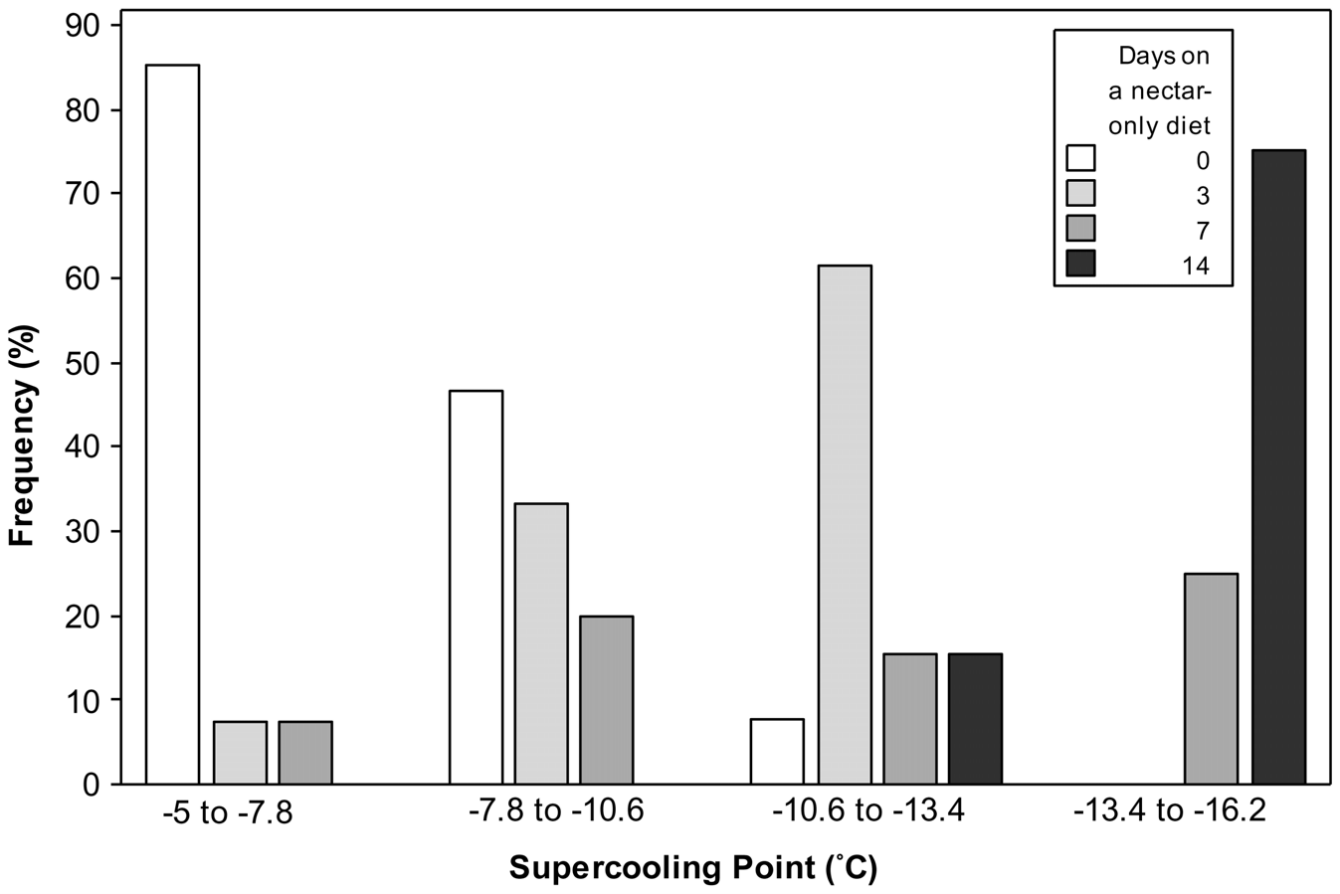
Frequency of bumblebee SCPs, partitioned into 4 equal groups, and their corresponding feeding regime. Frequency distributions of supercooling points from *Bombus terrestris audax* workers on a nectar-only diet for 3, 7 and 14 days and controls, *n* =  30, 14, 8 and 5 for controls, 3, 7 and 14 days of nectar feeding respectively.

A significant relationship was found between the number of pollen grains in the gut and supercooling point across all groups (Figure 6; R^2^ = 32.7%, *p*<0.01). Two highly erroneous outliers were removed, having a total of 104 and 18 pollen grains and corresponding supercooling points of −7.01 and −7.99°C respectively. The frequency of ‘high’ SCPs was clearly greater in controls and 3 day nectar-only fed samples, compared to 14 day nectar-only fed bees (Figure 7), with all 14 day nectar-fed bees having SCPs below −12.42°C. Neither controls nor 3 day bees were in this lowest SCP group. Additionally, 100% of 14 day nectar fed bees had no pollen in their guts (Table 2), compared to 57 and 25% for 3 and 7 days of nectar feeding respectively.

**Table 2:** Affect of nectar-feeding on the proportion of pollen grains in the gut of *Bombus terrestris audax*.

Days of nectar feeding	Proportion of bees with no pollen grains (%)
3	57
7	25
14	100

Proportions of *Bombus terrestris audax* workers with pollen grains in their guts after 3, 7 and 14 days of nectar-only feeding, *n* = 14, 8 and 5 for 3, 7 and 14 day nectar-fed workers respectively.

## Discussion

This study has identified that *B. t. audax* is a freeze avoiding species (*cf* [31]), with limited supercooling ability (mean SCP of workers −7.1±0.2 and queens −7.4±0.3°C). This is in contrast to several other bee species, e.g. *Osmia cornuta* and *O. rufa*, both with SCPs typically below −24°C [37], but is comparable with *Megachile rotundata* with SCPs of −8°C [38]. Despite this limited supercooling ability, both *B. t. audax* workers and queens are able to tolerate exposures to temperatures close to their SCP for short periods; for example, 80±16.3% survived after exposure to −5°C for 2 h (Figure 3).When subjected to acute (15 min) sub-zero cold stress, 90% of workers were able to survive at −5.0±1.1 and 50% at −7.8±1.1°C. Indeed, tolerance of sub-zero conditions is actually greater than this result might suggest, because the cooling rate of 0.2°Cmin^−1^ means bees were exposed to temperatures below 0°C for extended periods e.g. 65 mins below 0°C for a −5°C exposure. However, these temperatures represent thermal stresses that can occur across much of the native Northern European range of *B. t. audax* [45] during winter, so remaining active could have a significant impact on survival and thus the distribution and abundance of this species. Such is the case for *Nezara viridula* in Japan, whose northern expansion is limited by low January temperatures [46]. In this regard, it is important to note that it is higher autumn temperatures that most likely influence the physiological ‘decision’ for insects to avert diapause [9], and so become winter active. However, this does not preclude warmer winter conditions. Indeed, recent UK winters indicate an increasing frequency of extreme cold events [19].

Bees can of course behaviourally avoid the most extreme conditions by remaining within the subterranean colony. However, if a colony is to survive an entire winter period, workers cannot simply remain within the nest. At some point they must forage for nectar and pollen (potential sources described by [8]) to fuel colony development and the eventual establishment of the next generation of queens and males.

The ability of insects to rapidly cold harden allows them to mitigate the affect of sudden changes in temperature, e.g. during foraging, and fine tune their response to environmental temperature fluctuations [47]. *Bombus terrestris audax* clearly demonstrates an ability to undergo RCH (Figure 4) and, to our knowledge, this represents the first evidence of RCH in Hymenoptera. Interestingly, RCH was not found to lower the SCP of bumblebees, in common with other insects including the Antarctic midge, *Eretmoptera murphyi* [48], and house fly, *Musca domestica* [49]. RCH is especially relevant to winter activity because bees may need to forage more widely at a time of year when nectar and pollen resources are more limited. Equally, at certain threshold temperatures, activity will become restricted (*unpublished data*), meaning that individual bees may not always be able to return to the colony within the same 24 hour period, and thus be exposed to night time temperatures. Given that bumblebees are known to remain away from the colony at night [27] [28] and night time winter temperatures in recent years in the UK have reached well below −10°C [50] this may represent a significant survival risk to *B. t audax*.

In addition to acute cold exposure during foraging, winter active bumblebees are also likely to experience chronic low colony temperatures. While bumblebees do possess the ability to thermoregulate their colonies [24] [25], a lack of winter floral resources or an excessive thermoregulatory demand, may mean colonies are unable to consistently maintain a favourable temperature [51]. *Bombus terrestris audax* typically construct nests underground at a range of depths [52], and although this might buffer them from extreme air temperature fluctuations, there is still a survival risk. Recent (2009–2013) winter soil temperatures (10 cm depth) in the UK have consistently fallen below 5°C for many weeks, and below 0°C for periods of several days as far south as Rothamsted, Hertfordshire [53]. Commercially-produced colonies established outside and exposed to winter conditions in Birmingham (2012–13) did not survive to produce any new queens or males, and had very low levels of activity beyond November (*unpublished data*). Thus, winter activity caused by warmer autumn conditions disrupting diapause, could have devastating effects on the number of new queens establishing colonies in spring, with associated impacts on pollination service provision. This is not just relevant to *B. terrestris*. Other bumblebee species may have their diapause disrupted by climate change, and often have nests close to the surface or even at elevated sites, e.g. in bird boxes [54] where they could regularly be exposed to temperatures below −5°C, as well as freeze-thaw transitions.

The LTime50 for exposure to 0°C was 7.2±1.1 and 25.6±2.4 for workers and queens respectively. This suggests that active queens have a greater underlying cold tolerance than workers, even though the SCP of queens and workers were not significantly different. One explanation for this might be the more extensive fat reserves present in diapausing queens to facilitate winter survival and early spring foraging [55], and increased fat content is often correlated with enhanced cold tolerance [56]. However, it is not known if the fat content of active queens differs significantly from workers.

As hypothesised, manipulation of diet through the removal of pollen significantly decreased the SCPs of both workers and queens (Figure 5) with every ‘pollen free’ treatment having a significantly lower SCP than its ‘pollen-fed’ counterpart. This provides strong support for the ice-nucleating properties of pollen, in agreement with work [40] on *Megachile rotundata,* which showed increased freezing temperatures as a result of dietary ice nucleators. Diet has long been known to affect cold tolerance with Antarctic micro-arthropods shifting their SCPs from a summer ‘high’ group (mean SCP −7°C) to a winter ‘low’ group (mean SCP -25°C) after a period of starvation [36] and freeze avoiding insects evacuating their guts to exclude ice nucleating agents (INAs) [32]. To sustain winter active colonies, bumblebee workers are required to forage for pollen at low temperatures, therefore behavioural avoidance of ice nucleators is not possible. As a result, workers are at increased risk of freezing mortality when foraging which may lead to poor colony nutrition and death. This poses an unknown risk to winter active colonies at temperatures below −5°C, the highest SCP recorded in this study.

Recent harsh winters and an increasing frequency of extreme events pose significant threats to existing winter-active colonies. Coupled with a decrease in cold tolerance as a result of ice nucleation, foraging bumblebees are at risk of winter mortality which may lead to colony collapse. This is perhaps why sightings in winter are restricted to the southern UK. However, higher autumn temperatures, as a result of climate change, may facilitate winter-active bumblebee colonies in the future. This must be coupled with adequate floral resources, suitable nest sites and the successful production of new queens to establish colonies the following year.

The changing winter phenology of *B. t. audax* is typical of the current climate-change induced threats to pollinators, responsible for disrupting ecosystems and plant-pollinator interactions. In order to further understand this trend, future work must include quantification of these changes in a field setting, taking in to account activity and pollination behaviour, which is key to understanding the impact of climate change on pollinators.
